# Revascularization Enhances Walking Dynamics in Patients with Peripheral Artery Disease

**DOI:** 10.3390/applmech6020040

**Published:** 2025-10-02

**Authors:** Farahnaz Fallahtafti, Arash Mohammadzadeh Gonabadi, Kaeli Samson, Megan Woods, Iraklis Pipinos, Sara Myers

**Affiliations:** 1 Department of Biomechanics, University of Nebraska at Omaha, Omaha, NE 68182, USA; 2 Institute for Rehabilitation Science and Engineering, Madonna Rehabilitation Hospitals, Lincoln, NE 68506 USA; 3 Department of Biostatistics, University of Nebraska Medical Center, Omaha, NE 68198-4375, USA; 4 Department of Surgery, University of Nebraska Medical Center; 5 VA Nebraska-Western Iowa Health Care System, Department of Veterans Affairs, Omaha, NE 68105, USA

**Keywords:** Variability, Gait, Claudication, Kinematic, Nonlinear dynamics

## Abstract

Blocked or narrowed arteries restrict blood flow to the lower limbs, commonly leading to peripheral artery disease (PAD). Patients with PAD have been shown to have increased gait variability, which may contribute to higher rates of falls and worsen functional outcomes. Surgical revascularization seeks to restore blow blood to the legs, but it is unknown if this restoration enhances limb function. This study investigated whether gait variability changes in patients with PAD after revascularization surgery. Thirty-three patients with PAD exhibiting claudication symptoms were recruited for the study. Kinematic data were recorded using a motion capture system while the patients walked on a treadmill following a progressive treadmill protocol, both before and after undergoing revascularization surgery. Angular sagittal movements’ linear and nonlinear variability in the lower limbs were measured and compared before and after surgery across the ankle, knee, and hip joints. Following revascularization surgery, knee joint sample entropy (SampEn) decreased, suggesting improved gait regularity. Furthermore, the hip range of motion (ROM) significantly decreased, whereas the knee ROM significantly increased. The ankle joint showed significantly greater changes in the Lyapunov Exponent (LyE) relative to the pre-exercise condition compared to the hip and knee joints. No significant differences existed in the linear variability (standard deviation) of the ROM between joints. In individuals with PAD, revascularization surgery considerably increased knee ROM and gait regularity, indicating improved limb function and motor control. However, the ankle ROM remained unchanged, indicating the need for targeted strengthening exercises post-surgery.

## 1. Introduction

Peripheral artery disease (PAD) is characterized by limited blood flow to the limbs due to narrowed or blocked peripheral arteries ^1–4^. Blockages in the leg arteries prevent sufficient blood flow to working leg muscles during walking, which leads to ischemic pain known as intermittent claudication ^1–3, 5^. As individuals age, PAD becomes more common ^2, 6^. An increase in age, in addition to comorbidities like diabetes, tobacco use, and heart disease, may lead to an overall increase in the prevalence of PAD ^2, 4, 5^. Patients with PAD experience decreased mobility and poor health outcomes, including an increased risk of falls ^7^. Prior research found that patients with PAD exhibit decreased ankle and knee torque, power, and gait variability compared to older controls without PAD. These changes lead to increased energy expenditure, greater functional impairments ^8^, and a higher rate of falls as they age ^9^. These physical challenges significantly reduce the quality of life and independence and can elevate mortality rates ^10^.

Treatments for PAD include pharmacotherapy, exercise rehabilitation, and surgical revascularization ^11–14^. Pharmacotherapy interventions have moderate efficacy and are associated with improvement in maximal treadmill walking distance of only 15%-25% ^15^. Supervised exercise therapy effectively improves the distance patients can walk, however, not all patients’ walking capacity improves with supervised exercise therapy ^16^. Surgical revascularization for patients with PAD aims to restore hemodynamics, leading to improved tissue perfusion. Revascularization enhances gait, promotes wound healing, and preserves the patient’s ambulation ability ^11, 12^. PAD is the cause of 51% to 93% of all lower-leg amputations; therefore, revascularization treatment can be used to extend the lifetime of the limb affected by PAD ^17^. However, it is essential to accurately detect the effect of revascularization on the dynamic system of movement using advanced nonlinear techniques. The intrinsic movement patterns, characterized through nonlinear dynamics and variability metrics, are critical determinants of the system’s functional integrity and must be accurately identified to optimize therapeutic interventions. Revascularization has been shown to enhance tissue perfusion and gait mechanics, and it may further contribute to fall risk reduction by improving gait variability in individuals with PAD. Revascularization improves both tissue perfusion and gait, and it may also reduce fall risk by improving gait variability in patients with PAD.

Gait variability quantifies deviations across movement patterns and can be described as the stride-to-stride variations in kinematic measures that occur across multiple repetitions of a specific task, helping to distinguish gait alterations from healthy walking patterns ^18–21^. For example, while increased stride time variability can indicate potential gait abnormalities, complex fluctuations provide valuable insights into the underlying dynamics of the signal. Previous studies have shown that greater gait variability correlates with an elevated risk of falls and diminished functionality in older individuals ^18, 19^. Patients with PAD have more gait variability and nonlinear variability alterations at the ankle, knee, and hip joints compared to age-matched healthy controls ^10, 22^. Gait variability alterations exist in patients with PAD from the first step they take, even before the onset of claudication pain ^23^. Previous studies have determined that with successful surgical intervention, patients with PAD have less pain during activity, fewer leg ulcers, an overall improvement in gait, and increased quality of life ^11, 12, 24^. However, it has yet to be determined whether gait variability characteristics are restored after revascularization.

Although revascularization has been shown to reduce ischemia pain and enhance general gait mechanics in individuals with PAD, not much is known about how surgical intervention affects gait variability, an important indicator of motor control and fall risk. This study aimed to determine changes in gait variability in patients with PAD after surgical intervention because it can reveal necessary information about improvement in function following revascularization. We hypothesize that revascularization treatment will improve movement stability and reduce variability in lower limb joint kinematics during treadmill walking in patients with PAD. Specifically, we expected that the largest Lyapunov exponent (LyE), sample entropy (SampEn), and standard deviation (std) of joint angles would decrease, indicating greater movement regularity and stability. The results of this study can benefit therapists by indicating post-revascularization improvement, potential impacts of fall risk and function, and deciding whether companion therapies would benefit patients after revascularization surgery.

## 2. Materials and Methods

### 2.1. Participants

Patients with PAD were invited to participate in vascular surgery clinic visits at the Nebraska-Western Iowa Veteran Affairs Medical Center and the University of Nebraska Medical Center. The Internal Review Boards of the University of Nebraska Medical Center and the Nebraska Western Iowa Veteran Affairs Medical Center approved this study. Before participating, each participant provided informed consent. A history of chronic claudication, exercise limitation, a brachial ankle index of less than 0.90 at rest, and no sign of any neurological or muscular symptoms that would limit walking were the criteria for including subjects in this study. Potential subjects were all individuals who had decided to undergo surgical revascularization for PAD. Patients without PAD, those with PAD-related rest discomfort, or those with exercise restrictions unrelated to claudication were excluded from the study.

### 2.2. Data Collection

This study compared changes in gait patterns in patients with PAD at baseline and six months post-revascularization. Upon arriving at the laboratory, participants changed into form-fitting suits to prepare for data collection. Next, thirty-three retro-reflective markers were placed on specific anatomical locations of the patient’s lower extremities ^25^. We used a 12-camera motion capture system (Motion Analysis Corporation, Rohnert Park, CA) operating at 60 Hz to record the three-dimensional trajectories of these markers. Patients walked on a treadmill at a self-selected comfortable speed. Participants completed at least five walking trials to ensure reliable data capture.

Patients completed a Gardner progressive treadmill test, which included 2 mph walking with a 0% incline that increased by 2% every 2 minutes. When the patients first experienced pain, they informed the person operating the treadmill but continued walking until claudication forced patients to stop walking. This procedure was conducted both before and after the patient’s revascularization surgery.

### 2.3. Data Analysis

Marker data were tracked, but no filter was applied to smooth the marker data to increase the accuracy of capturing minor variations in joint motion ^26, 27^. While no filtering was applied to maintain small variations in joint movement, we recognize the potential for noise interference. To reduce noise, we ensured standardized marker placement, consistent walking conditions, and high-quality motion capture tracking. All trials were cropped at 3500 data points, which included at least 30 strides of data. Visual3D was used to calculate the lower limbs’ sagittal plane joint angles. The average and std of the ROM data were calculated for the ankle, knee, and hip as linear measures of variability. Linear measures, such as the average and std of the ROM, provide a basic understanding of joint mobility and variability. However, they do not capture the intrinsic behavior of motor control adaptations following revascularization. Nonlinear measures, including SampEn and the LyE, offer a deeper understanding of gait dynamics by evaluating movement regularity, adaptability, and stability. Nonlinear measures used in this study were SampEn and the LyE. Nonlinear analysis was calculated for the time series of hip, knee, and ankle joint angles. SampEn estimates whether patterns are likely to repeat themselves ^28, 29^, which can predict gait patterns and indicate regularity. Entropy values near zero indicate a highly regular motion pattern, and larger values indicate irregularity. Knowing the regularity of gait data using SampEn is essential because it provides insights into the health and function of the neuromuscular control systems ^30^, helps in the early detection and monitoring of gait abnormalities and neurological conditions ^31^, and can help refine rehabilitation programs by assessing dynamic changes in gait patterns. High regularity in gait signals is considered rigid and periodic, while excessive irregularity indicates loss of coordination; both patterns exist in pathological gait signals ^32^. A vector length (m) of 2 and a tolerance (r) of 0.2 times the std of the time series were chosen for analysis of SampEn ^33^. The most prominent LyE was used to determine the deviation of signal patterns from steady state ^34^. LyE data quantifies the ability to react and adapt to movement disturbances ^34^. This is useful because it is known that patients with PAD usually have less of an ability to adapt to changing conditions or disturbances based on prior research ^10, 35^. Smaller values of the LyE indicate a system with slight divergence ^36^. The largest LyE measures the maximum separation of close trajectories of a dynamical system in a particular phase space dimension ^36^. A custom MATLAB code calculated the largest LyE and SampEn for each joint angle time series (The MathWorks, Inc., Natick, MA).

### 2.4. Statistics

This study is a sub-analysis of previously collected data, and the sample size was determined based on the primary study’s power analysis. The original power analysis used gait outcomes as the main variable rather than variability measures. While the current sub-analysis focuses on kinematic variability, the sample size was not specifically determined for these measures. However, given the existing dataset, we aimed to explore meaningful trends in nonlinear and linear kinematic metrics within the available sample. Changes in linear and nonlinear measurements pre- and post-surgery were calculated by subtracting the pre-values from the post-values. Descriptive statistics are given as means and std (Table 2). For each measure, a general estimating equation, with change in measurement as the outcome, was used to assess differences in change between joints. To assess change over time for a given joint, model-estimated means were assessed at the mean value for the pre-surgical measurement for that specific joint and were compared to a value of zero (indicating no change). To assess differences in observed change between joints, comparisons between joints were made while holding the pre-value measurement constant. Model estimates are given with Bonferroni-adjusted 95% confidence intervals, and p-values for multiple comparisons were Bonferroni-adjusted. This trade-off increases the likelihood of Type II errors. Future studies may consider alternative multiple comparison adjustments, such as the false discovery rate, which offers a more flexible approach while maintaining error control. To help visualize model results, the raw data was plotted with vertical box plots and displayed with effect sizes of the change between pre and post time points, which were calculated for each joint by dividing the average change score by the std of those change scores. To account for running all statistical tests four times, once for each outcome of interest, we used a more conservative alpha level of 0.0125 to determine statistical significance. All analyses were performed using SAS software version 9.4 (SAS Institute Inc., Cary, NC).

## 3. Results

Thirty-three patients with PAD completed this study (Table 1), which provides the demographic characteristics of all participants who attended pre- and post-visits. Table 2 shows the values for all outcome variables before and after revascularization associated with ankle, knee, and hip.

Results of the within-joint comparison showed a significant decrease in the SampEn values for the knee joint from pre- to post-surgery, with an estimated mean change of −0.018 (95% CI: −0.037 to 0.000, p = 0.05). The changes in SampEn for the knee joint were significantly larger than that for the hip or ankle (p < 0.001; Table 3). Furthermore, the changes in the largest LyE values for the ankle joint were significantly larger changes than hip or knee (p < 0.003).

**Figure 2.**
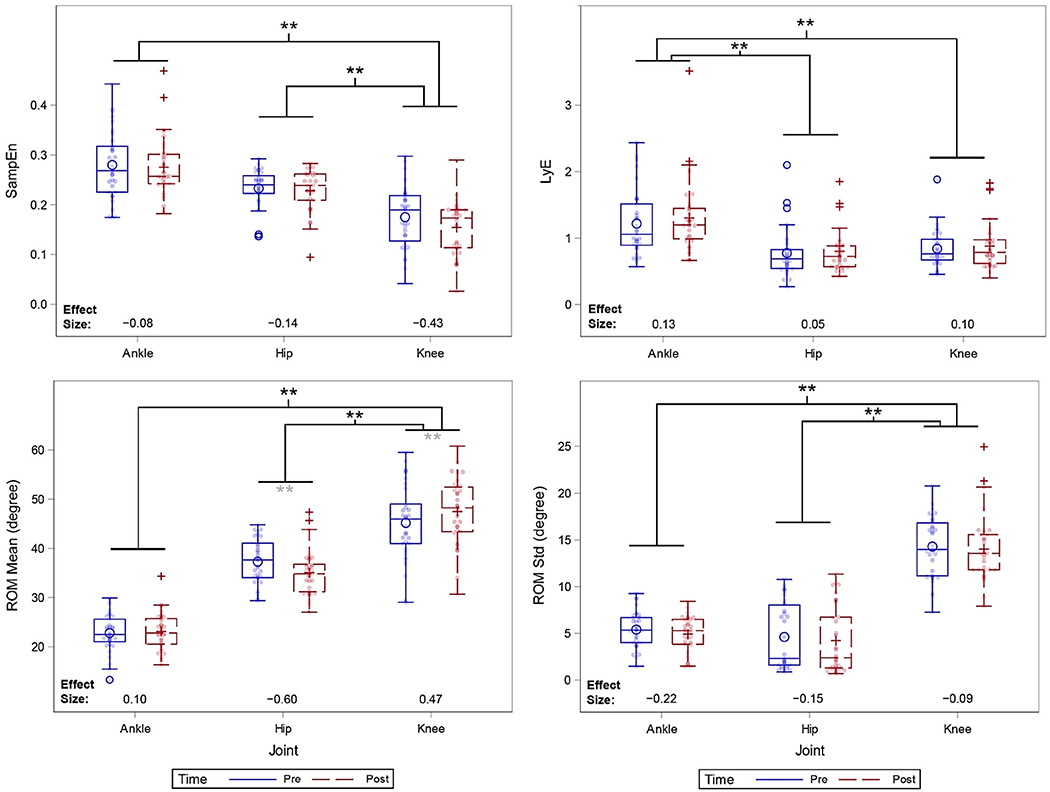
Raw values for each of the outcome measures by joint and time point. Effect sizes were calculated by dividing the change scores (i.e., post – pre) within a joint by the std of those change scores. Double asterisks indicate p < 0.01, where p-values are derived from the model in Table 3. Grey asterisks indicate significant change (after adjusting for baseline value) between the pre and post time points; black asterisks indicate significant differences between joints in model estimated pre-to-post change (i.e., the change between pre and post in one joint significantly differed from the change in another joint).

There was a significant increase in the ROM values for the knee joint from pre- to post-surgery, with an estimated mean increase of 2.322 (95% CI: 0.748, 3.896, p = 0.002). Conversely, there was a significant decrease in the mean ROM values for the hip joint from pre- to post-surgery with an estimated mean decrease of −2.232 (95% CI: −3.806, −0.657, p = 0.003). The model-estimated change in ROM mean for the knee joint was significantly larger than that for the hip or ankle (p < 0.001) (Table 3, Figure 2).

For the linear variability (std) of ROM, the differences across joints were not significant in pairwise comparisons. Additionally, the pre-to-post measures within each joint did not demonstrate any significant changes.

In addition to these statistical findings, the alterations in joint kinematics have potential clinical implications for stair climbing and overall gait stability.

## 4. Discussion

Intermittent claudication results in limited walking and exercise performance, which affects the overall quality of life in patients with PAD. In patients who are significantly impaired in their activities of daily life, surgical intervention (revascularization) is an appropriate treatment ^37^. While clinicians are generally devoted to describing the average behavior of gait characteristics, comparing the gait variability before and after treatment could be informative of whether function is restored. The amount and structure of gait fluctuations indicates the motor control of gait ^38^. This study tested whether surgical intervention impacts linear and nonlinear kinematic measures of lower limb joint angle motion during treadmill walking in patients with PAD. We hypothesized that the largest LyE, SampEn, and std of joint angles would decrease after revascularization treatment while the ROM would increase after revascularization. Our results partially supported our hypothesis, where regularity decreased, and ROM increased after surgery in the knee joint, while other variability measures remained constant and hip ROM decreased. The findings present a combination of advantages and disadvantages. On one hand, an increased Knee ROM and regularity is consistent with gait performance seen in healthy individuals and suggests an improvement in motor control and efficiency. This would indicate better neuromuscular coordination, which could lead to increased walking performance, a lower risk of falls, and easier everyday tasks. On the other hand, the associated reduction in hip joint ROM may limit stride length and the ability to adjust to perturbations. This suggests that although revascularization can restore some gait characteristics, targeted rehabilitation would be required to maintain hip mobility.

In support of our hypothesis, we observed more regular behavior after revascularization in knee joint angle motion. This decrease in SampEn from pre- to post-surgical values in the knee joint represents a more regular motion pattern in the joint angle time series. A more regular pattern may indicate increased postural control’s “efficiency” or “automaticity” following revascularization^39^. This positive change from the surgical intervention aligns with our previous findings, demonstrating that supervised exercise therapy can enhance the regularity of lower limb joint motion ^33^. Knee joint SampEn was larger than hip and ankle (Table 2). Studies have demonstrated that patients with PAD and intermittent claudication exhibit increased variability in knee joint motion during walking ^22^. The knee joint plays a central role in locomotion and bears significant stress and load during walking. Improved blood flow from the revascularization can have a more immediate and noticeable effect on its function. Furthermore, the knee has a larger ROM compared to the ankle and hip, which could result in more detectable improvement post-surgery.

According to the results of the current study, the largest LyE values were not significantly different before and after revascularization in each separate joint. The absence of significant changes in the largest LyE may be attributed to the chronic nature of PAD, wherein a six-month intervention or surgical treatment alone may not be sufficient to restore coordination and gait control. PAD is associated with chronic neuromuscular impairments, including muscle atrophy, which may persist even after revascularization. These chronic neuromuscular damages may limit the extent of immediate improvements in the largest LyE. PAD induces enduring alterations in gait patterns, and reversing these changes likely requires extended periods of rehabilitation and comprehensive therapeutic strategies. Our findings indicate that the largest LyE for the ankle joint exhibited greater changes from pre- to post-intervention compared to the hip and knee joints, consistent with previous research highlighting increased variability in the ankle joint ^33^. This indicates variability may reflect persistent muscle impairments, such as adverse conditions in the calf muscles of these patients ^40^. Similar to populations undergoing heart or orthopedic surgeries, where prolonged recovery and additional rehabilitation are essential for meaningful improvements, PAD patients may also benefit from extended and multifaceted rehabilitation programs to effectively enhance gait dynamics and coordination. Therefore, the limited changes observed in our study likely reflect the entrenched gait impairments associated with PAD and underscore the necessity for prolonged rehabilitation efforts to achieve significant functional recovery.

Nonlinear analysis enables us to understand the biological system changes across time series ^41^. On the other hand, linear analysis helps us understand the magnitude of variability at specific points in the time series ^41, 42^. Although the linear variability in lower limb joint angle motion remained unchanged, the ROM changes were observed in the absolute mean values of knee and hip before and after revascularization. Knee ROM mean values were increased from 45.203 (pre-surgery) to 47.524 (post-surgery), and hip ROM mean decreased from pre- to post-surgical analysis from 37.306 to 35.076 (Table 2). These joint-specific shifts support prior findings that surgery intervention may not translate into immediate functional improvements in daily activities^43, 44^, highlighting the need for additional rehabilitation strategies.

In our prior study, patients with PAD showed hip and knee ROM that was not significantly different from healthy participants ^45^. Calf muscle deficiencies in patients with PAD could affect knee motion and physical function during walking. Previous works evaluating the histomorphology and biochemistry of the calf muscles has demonstrated the presence of an ischemic myopathy in the legs of PAD patients ^46, 47^, which may not be enhanced right after revascularization. According to the results of this study, we did not observe improvement in the ankle ROM, which could be due to unchanged muscle strength in patients with PAD after revascularization. Therefore, strengthening exercises could be recommended to increase the recovery of ankle ROM after revascularization. The weakness of the ankle musculature following revascularization may also result in increased energy for walking. Future rehabilitation programs should incorporate targeted techniques to improve ankle function, such as progressive resistance training like heel raises and seated calf presses, and improve neuromuscular control through proprioceptive training, including balance board exercises. Therefore, patients may benefit from enhanced ankle function, lower walking energy costs, and eventually better long-term gait outcomes after revascularization.

This study did not come without limitations. This study did not capture self-reported pain or fatigue and should be considered in future studies. Also, this secondary analysis included 33 participants and was potentially underpowered for the present gait-biomechanics endpoints. Consequently, subgroup and exploratory findings should be interpreted with caution. Furthermore, while we carefully documented medication use, it was not possible to control or isolate the effects of these therapies. Therefore, the potential influence of pharmacological treatments on the study outcomes is acknowledged as a limitation. While some lifestyle information was collected when available, these factors were not consistently controlled or analyzed. Patients with PAD typically have limited walking distances, which can influence nonlinear measures, as more extended datasets are generally more effective for identifying the dynamics of gait patterns. Future simulations may be beneficial in complementing these findings. Complementary methods, such as artificial neural networks^48^, could also be utilized to model nonlinear gait characteristics and predict physiological outcomes in clinical populations such as patients with PAD. It is important to note that each joint’s pre-revascularization ranges of outcome variables did not always significantly overlap. Future studies could also compare muscle oxygenation and muscle pathology of the calf muscle in patients with PAD to determine how muscle activity is altered with and without surgical intervention.

## 5. Conclusions

The study concludes that revascularization could improve gait regularity in individuals with PAD, as evidenced by decreased SampEn in knee joint motion. This indicates that restored blood flow may be promising for improving motor control. Improved joint function following revascularization was also shown by increased knee ROM. However, an unchanged ankle ROM could indicate that revascularization alone does not restore calf muscle deficiencies in PAD. We conclude that revascularization alone does not completely restore gait function in patients with PAD. Decreased hip ROM suggests the importance of post-physical therapy measures. Therapists should consider combining mobility-focused therapy with gait retraining to improve long-term functional results. Future work should study whether rehabilitation, such as strength training or walking exercises, can compensate for reduced hip ROM while preserving the improvements in motor control.

## Figures and Tables

**Figure 1.**
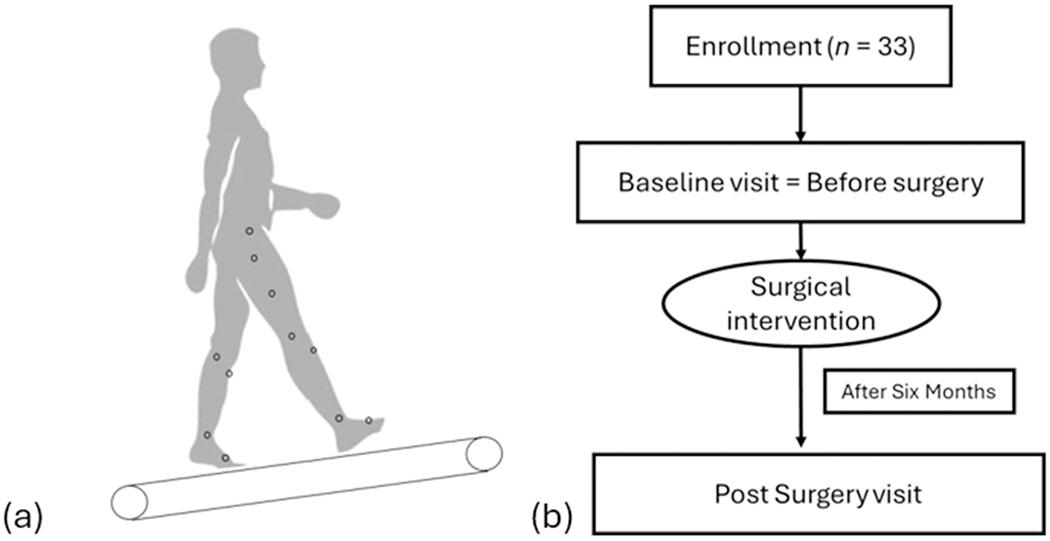
Experimental apparatus and study timeline. **(a) Experimental set-up.** Participants walked on an instrumented treadmill. Thirty-three retro-reflective markers were attached to lower-extremity landmarks, and a 12-camera motion-capture system sampled three-dimensional trajectories. **(b) Study design.** Patients with PAD completed two gait assessment sessions: one prior to surgical revascularization (Pre) and one six months post-surgery (Post).

**Table 1.** Demographic data for the participants (n=33) who completed both visits to the study. All values are written as mean (std).

Age (years)	Body mass (kg)	Height (m)	BMI ^1^(kg/m2)
62.70 (5.31)	85.76 (14.96)	1.76 (0.07)	27.46 (4.12)

1 BMI = Body Mass Index

**Table 2.** Mean ± std of linear and nonlinear kinematic measures for the ankle, knee, and hip joints for pre-and post-revascularization

Group	Ankle	Knee	Hip
**ROM Mean (degrees)**			
Pre-surgical	22.781 ± 3.841	45.203 ± 7.672	37.306 ± 4.583
Post-surgical	23.122 ± 3.775	47.524 ± 6.806	35.076 ± 4.539

**ROM Std (degrees)**			
Pre-surgical	5.404 ± 1.940	14.296 ± 3.245	4.616 ± 3.526
Post-surgical	4.926 ± 1.932	14.030 ± 3.519	4.234 ± 3.547

**SampEn**			
Pre-surgical	0.280 ± 0.068	0.175 ± 0.062	0.233 ± 0.039
Post-surgical	0.275 ± 0.061	0.154 ± 0.054	0.228 ± 0.043

**LyE**			
Pre-surgical	1.218 ± 0.500	0.839 ± 0.286	0.773 ± 0.371
Post-surgical	1.304 ± 0.546	0.879 ± 0.348	0.800 ± 0.310

**Table 3.** Statistical comparisons were conducted for each joint before and after surgical intervention (within joint measurement), and the changes between joints from the pre-intervention condition were also analyzed. *P*-values were derived from linear mixed models, with separate models run for each dependent variable.

Outcome	Mean value before surgery	Model Estimated Mean Change in Outcome	Bonferroni Adjusted Confidence Interval for Mean Change		Bonferroni Adjusted *P*-value for Change Within Each Joint	*P*-value for Main Effect of Joint (Differences in Change Observed Between Joints)
**SampEn**						<0.001
Ankle	0.28	−0.005	−0.023	0.014	1.00 ^k^	
Hip	0.23	−0.003	−0.022	0.015	1.00 ^k^	
Knee	0.17	−0.018	−0.037	0.000	0.05 ^a,h^	

**LyE**						0.003
Ankle	1.22	0.084	−0.152	0.321	1.00 ^h, k^	
Hip	0.77	0.029	−0.111	0.170	1.00 ^a^	
Knee	0.84	0.039	−0.112	0.189	1.00 ^a^	

**ROM Mean (degree)**						<0.001
Ankle	22.78	0.341	−1.233	22.78	1.00 ^k^	
Hip	37.31	−2.232	−3.806	37.31	0.003 ^k^	
Knee	45.20	2.322	0.748	45.20	0.002 ^a,h^	

**ROM Std (degree)**						<0.001
Ankle	5.40	−0.476	−1.486	0.535	0.75 ^k^	
Hip	4.62	−0.384	−1.394	0.627	1.00 ^k^	
Knee	14.30	−0.268	−1.278	0.743	1.00 ^a,h^	

All models adjusted for the pre-value associated with the outcome. The model estimated mean changes to be calculated at the mean pre-value for each joint. Differences between joints were assessed, assuming the pre-value is constant across joints.

a Significantly differs from the ankle (Bonferroni adjusted *p*-value for post hoc pairwise comparison < 0.01).

h Significantly differs from the hip (Bonferroni adjusted *p*-value for post hoc pairwise comparison < 0.01).

k Significantly differs from the knee (Bonferroni adjusted p-value for post hoc pairwise comparison < 0.01).

## Data Availability

The raw data in this study is available upon request from the authors.
